# Phototheranostics Using Erythrocyte-Based Particles

**DOI:** 10.3390/biom11050729

**Published:** 2021-05-13

**Authors:** Taylor Hanley, Raviraj Vankayala, Chi-Hua Lee, Jack C. Tang, Joshua M. Burns, Bahman Anvari

**Affiliations:** 1 Department of Bioengineering, University of California, Riverside, CA 92521, USA; thanl001@ucr.edu (T.H.); vankayalaraviraj999@gmail.com (R.V.); jtang014@ucr.edu (J.C.T.); jburn006@ucr.edu (J.M.B.); 2 Radoptics, Limited Liability Company, 1002 Health Sciences Road, East, Suite P214, Irvine, CA 92612, USA; 3 Department of Biochemistry, University of California, Riverside, CA 92521, USA; clee188@ucr.edu

**Keywords:** delivery systems, red blood cells, photothermal therapy, photodynamic therapy, cancer, imaging

## Abstract

There has been a recent increase in the development of delivery systems based on red blood cells (RBCs) for light-mediated imaging and therapeutic applications. These constructs are able to take advantage of the immune evasion properties of the RBC, while the addition of an optical cargo allows the particles to be activated by light for a number of promising applications. Here, we review some of the common fabrication methods to engineer these constructs. We also present some of the current light-based applications with potential for clinical translation, and offer some insight into future directions in this exciting field.

## 1. Introduction

In recent decades, numerous types of constructs have been developed for the delivery of therapeutic and imaging payloads to target sites of interest. The overarching goals of engineered delivery systems are to protect the payload from non-specific interactions with biomolecules and other molecular entities in the milieu of physiological environments, and minimize clearance by the immune system so that sufficient amounts of the payload can reach the target site to achieve the desired efficacy without inducing non-specific toxicity and reducing harmful off-target side effects [1].

A contemporary approach in engineering of delivery systems is based on the use of cells or cell-derived constructs to encapsulate the desired payload, or cell membranes to coat the payload. Examples include delivery systems based on macrophages, lymphocytes, neutrophils, and stems cells [2,3]. A fundamental principle underlying the use of cell-based or cell-derived systems is that the built-in mosaic of the cell membrane can provide the camouflaging machinery to protect the payload from recognition by immune cells and increase the bioavailability of the payload.

Red blood cells (RBCs) (erythrocytes) are a particularly promising delivery system due to the presence of membrane proteins including CD47, which prevents phagocytic uptake by macrophages, as well as decay-accelerating factor (DAF) (CD55) and CD59, which protect the RBCs from complement damage and cell lysis by preventing the formation of membrane-attack complexes [4]. The presence of these “self-markers” on RBCs has the potential to increase the circulation of a desired payload embedded within an RBC-based construct as a result of protecting the payload from recognition and subsequent removal or destruction by the immune system. One of the earliest examples of RBCs being used as carriers dates back to 1973, when Ihler et al. demonstrated the successful loading of RBCs with various enzymes [5]. There has been a significant increase in RBC-derived constructs in recent years, especially in the field of nanomedicine, where RBCs have reportedly been used as carriers for not only enzymes, but antibiotics, nanoparticles (NPs), and various drugs as well [6]. More recently, RBC-based payload delivery systems have been extensively reviewed [4,6,7,8,9,10,11,12], and new sub-fields, such as systems designed for the delivery of cancer therapeutics [13] and vascular imaging [14], are emerging. A particular use of RBC-derived constructs is in relation to loading of optical cargos such as quantum dots (QDs) [15,16,17], metallic materials [18,19,20], and organic molecules [21,22,23], in order to use the constructs for optical imaging and sensing, and phototherapeutic applications. This review specifically covers the development of RBC-derived constructs for light-based theranostics and summarizes some of the current potential clinical applications for these constructs. We provide a listing of the acronyms used in this manuscript in Table 1.

## 2. Fabrication of RBC-Derived Constructs

### 2.1. Substrate Coating by RBC Membranes

A majority of light-based RBC-derived constructs involve using the RBC membrane as a coating for a cargo in order to endow the cargo with the immune evasion properties of a native RBC. RBC coatings have been used for a number of optical cargos such as various metals and semiconductors. For example, Ren et al. coated iron oxide (Fe_3_O_4_) magnetic nanoclusters with RBC membranes for photothermal therapy (PTT) [24]. Gold NPs, such as gold nanorods fabricated by Li et al., have also been coated with RBC membranes for use in PTT [25]. Semiconducting materials, such as the MoSe_2_ nanosheets designed by He et al., were similarly coated with RBC membranes, again for PTT [26]. NPs made from organic molecules have also been coated with RBC membranes. For example, Ye et al. coated spherical NPs comprised of chemodrug 10-hydroxycamptothecin (HCPT) and the near-infrared (NIR) dye indocyanine green (ICG) with RBC membranes, and used them for combined chemotherapy and PTT [27]. Chen et al. prepared hollow mesoporous Prussian blue (PB) NPs loaded with the chemotherapeutic drug doxorubicin (DOX), and coated them with RBC membranes for combined PTT and chemotherapy [28].

Another common optical cargo is upconversion nanoparticles (UCNPs). These NPs are usually made from rare-earth elements and are capable of converting longer wavelengths of light, such as NIR wavelengths, into shorter wavelengths, such as visible or UV light [29]. For example, Ding et al. prepared NaYF_4_:Yb/Er UCNPs coated with RBC membranes and containing the photosensitizer merocyanine 540 (MC540) [30]. Here, the UCNPs excited by 980 nm laser light resulted in luminescence in the 530–550 nm range, which was able to excite the MC540 for photodynamic therapy (PDT).

The examples mentioned thus far have involved coating a NP made from a material capable of interacting with light. However, there are some instances where NPs themselves are loaded with an optical cargo before being coated with an RBC membrane. For example, Yang et al. fabricated triblock copolymer nanoparticles loaded with the dye IR780 for phototherapy and docetaxel (DTX) for chemotherapy [31]. These particles were then coated with RBC membranes for enhanced circulation. In addition, there are some constructs where the RBC membrane is used to coat a non-optical cargo, and the optical cargo is incorporated into the construct using other methods. For example, Su et al. loaded polymeric NPs with paclitaxel for chemotherapy, and then coated the particles with RBC membranes that had the cyanine dye 1, 1-dioctadecyl-3, 3, 3, 3-tetramethylindotricarbocyanine iodide (DiR) inserted into the lipid bilayer for PTT [32]. Here, the optical cargo is not in the core of the construct, but is instead in the membrane coating itself.

**Figure 1:**
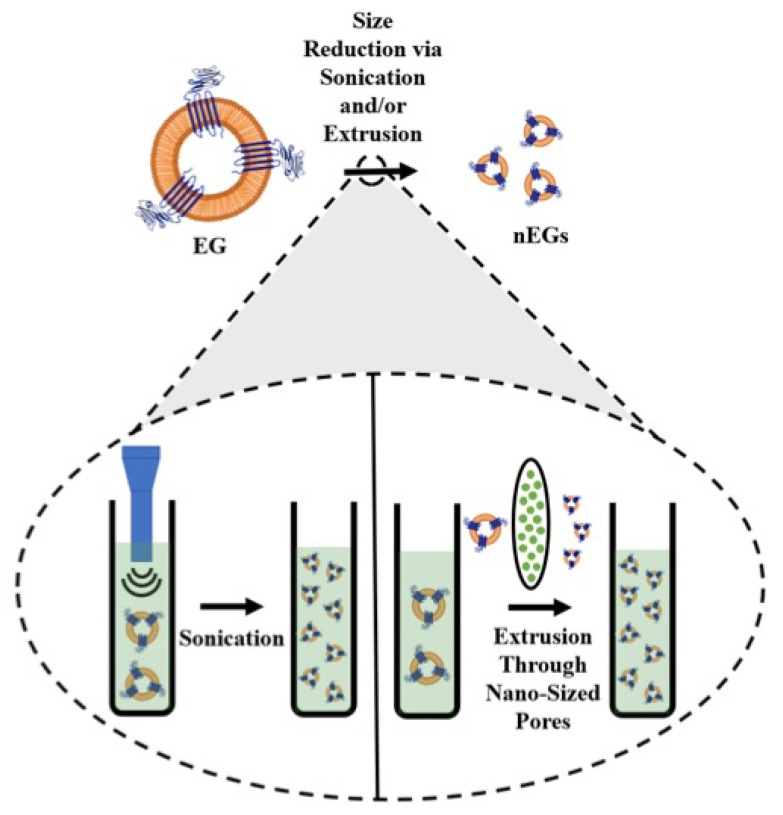
Schematic illustration of RBC membrane coating. RBCs are depleted of their hemoglobin using a hypotonic treatment to form erythrocyte ghosts (EGs). For illustration purposes, CD47 is shown as one of the RBC transmembrane proteins retained on EGs. The inset illustrates the sonication and extrusion size reduction methods. During sonication, sound waves are applied to EGs, while during extrusion, the EGs are pushed through filters with nano-sized pores, both of which result in formation of nEGs.

All of these examples show that there are a large number of constructs that can be coated with RBC membranes. In order to coat a cargo with the RBC membrane, whole blood is first washed with an isotonic buffer such as phosphate-buffered saline (PBS) to isolate the RBCs. The isolated RBCs are treated with a hypotonic buffer to deplete hemoglobin from the RBCs, resulting in erythrocyte ghosts (EGs). The EGs can then be scaled from the micro- to the nano-sized dimensions. Common methods for size reduction are mechanical extrusion, sonication, or a combination of the two. During mechanical extrusion, EGs are pushed through membranes with pores of a predetermined size, usually on the order of 200–800 nm. This leads to membrane lysis and reformation into smaller vesicles [33]. Sonication, or the application of sound waves with varying frequencies, is another cell disruption method [34]. Sonicating EGs also results in membrane rupture, and the subsequent reformation of nano-sized membranes [35]. A schematic illustrating the steps for formation of nano-sized EGs (nEGs) is shown in Figure 1.

**Figure 2:**
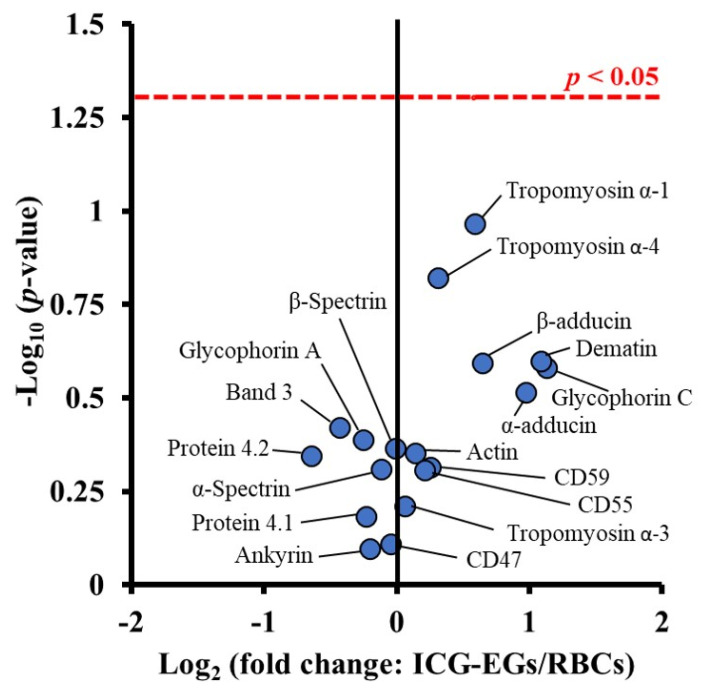
Volcano plot associated with proteomics analysis of human RBCs, and ICG-EGs. Each dot represents a particular protein. There are no statistically significant differences in the ratios of ICG-EGs proteins to RBCs proteins located below the red dashed line.

Since both sonication and extrusion result in membrane rupture, they can be used not only to reduce the change EGs to nEGs, but also to facilitate coating of an optical cargo. Mixing nEGs with a NP and repeating the sonication and/or extrusion facilitates the coating of the cargo with the RBC membrane, resulting in a construct with a core-shell morphology where the NP is coated by a membrane layer. Transmission electron microscope (TEM) images show that the RBC membrane conforms to the shape of the coated substrate. This has been shown in the work done by Rao et al., where coating hexagonal UCNPs with RBC membranes resulted in a hexagonal construct for cancer imaging [36]. Wang et al. have also developed spherical bovine serum albumin (BSA) nanoconstructs that encapsulate ICG and the chemodrug gambogic acid, and when coated in RBC membranes also result in a spherical nanoconstruct for chemotherapy and PTT [37]. These examples show a uniform membrane coating on a NP core. In addition to confirming the membrane coating using imaging, RBC membrane coatings have been confirmed by SDS-PAGE [36,38,39,40], as well as Western blots demonstrating the presence of CD47 on the final construct [31,41]. Our proteomics analysis based on tandem mass spectrometry confirm that CD47, CD55, CD59 and other membrane and cytoskeletal proteins including α-spectrin are retained in EGs loaded with ICG (ICG-EGs) (Figure 2).

Constructs that employ RBC membranes as a substrate coating have also been shown to be stable, with many constructs showing minimal changes in size after storage of up to one week [42,43]. We have demonstrated that after 12 h of storage in the dark at 4 and 37 °C, fluorescence emission of nEGs loaded with ICG (ICG-nEGs) was retained, whereas there was nearly a 40% reduction in the emission for free ICG [44]. The zeta-potentials of ICG-EGs and ICG-nEGs formed by extrusion were ~ −12.8 mV, and not significantly different from that for RBCs [44], indicating that the carboxyl groups of sialoglycoproteins, which are associated with much of the negative charge of RBCs, were retained during the fabrication of the particles. We have also found that there is only approximately a 5% reduction in ICG monomer absorbance of ICG after 8 days of storage in isotonic PBS at 4 °C in the dark [45]. We have determined that only approximately 5% of ICG leaks from ICG-nEGs over 48 h of storage at 37 °C [46]. We have also investigated the optical and physical properties of ICG-nEGs stored at −20 °C for up to 8 weeks and then thawed at room temperature [47]. Our results showed that the hydrodynamic diameter, zeta-potential, absorbance, and NIR fluorescence emission of ICG-nEGs were retained following the freeze–thaw cycle. The ability of ICG-nEGs in NIR fluorescence imaging of ovarian cancer cells, as well as their biodistribution in reticuloendothelial organs of healthy Swiss Webster mice after the freeze–thaw cycle were similar to those for freshly prepared ICG-nEGs.

While most of the previous examples have involved constructs where the RBC membranes coat a single NP, there are exceptions. For instance, Liu et al. encapsulated ICG in anionic BSA nanoclusters to form complexes with the chemotherapeutic 1,2-diaminocyclohexane-platinum (II) (DACHPt) [48]. These nanoclusters were coated with RBC membranes, resulting in constructs with an outer membrane shell and containing multiple BSA-based nanoclusters inside. In addition, researchers have shown that RBC membrane coatings are not limited to NPs. Gao et al. designed microparticles comprised of RBC-shaped magnetic hemoglobin containing Fe_3_O_4_ NPs and ICG, as magnetically-navigating PDT mediators [49].

### 2.2. Constructs That Use the RBC As a Carrier

While most light-based RBC-derived constructs use the RBC membrane as a substrate coating, there are a growing number of constructs that use the RBC as a carrier. Constructs that use the RBC as a carrier can have a variety of optical cargos, including organic molecules [50,51,52] and NPs made from various metals [18] or semiconductors [53,54]. While constructs that use the RBC membrane as a coating primarily use sonication and/or extrusion to facilitate substrate coating, there are a number of different loading methods for constructs that use the RBC as a carrier. Loading methods can be applied directly to RBCs, as well as EGs and nEGs. However, the vast majority of RBC-derived carriers are fabricated from RBCs. These methods can be broadly divided into those that load a cargo to the interior of the carrier, and those that load the cargo to the exterior of the construct.

#### 2.2.1. Methods for Interior Loading of an Optical Cargo

##### Hypotonic Loading

**Figure 3:**
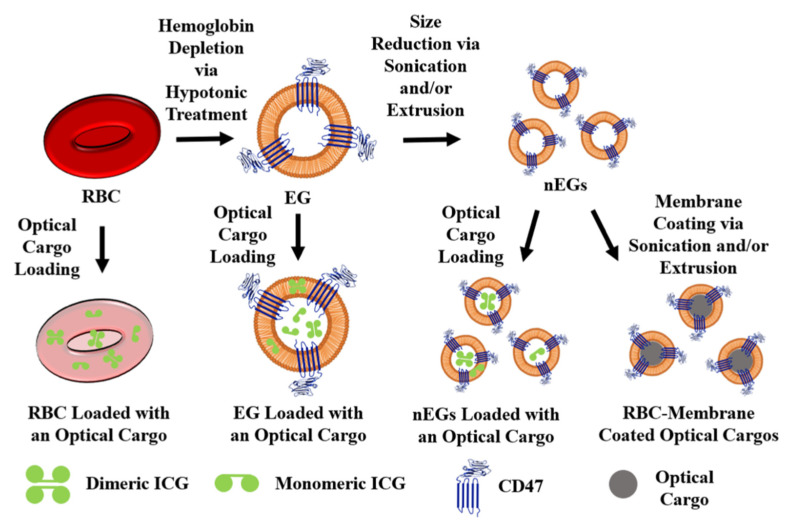
Schematic illustration of RBCs, EGs, and nEGs loaded with an optical cargo to serve as RBC-derived carriers. For illustration purposes CD47 is shown as one of the RBC transmembrane proteins retained on EGs. A representative spherical nanoparticle, to be coated with RBC membrane, is shown as the optical cargo for constructs formed using sonication and/or extrusion. Indocyanine green (ICG) is shown in its dimeric and monomeric forms as a representative optical cargo.

The most common loading methods for RBC carrier constructs are those that involve hypotonic buffers. When RBCs are placed in a hypotonic buffer they swell, causing the formation of pores for removal of intracellular components, but also allowing the internal loading of the cargo [50]. For example, Wu et al., using a hypotonic treatment, were able to simultaneously load RBCs with DOX, magnetic NPs, and QDs capable of fluorescence emission [15]. We have used a similar method to load EGs with ICG using Sorenson’s buffer, resulting in micron-sized light-responsive constructs [46]. Our group has also applied this method to nEGs, where we first extruded EGs to form nEGs before incubating them with Sorenson’s buffer and ICG, resulting in the loading of ICG into the nEGs [46]. Therefore, hypotonic loading methods can be applied to RBCs, EGs, and nEGs. RBCs can also be loaded using hypotonic dialysis. Here, the RBCs are typically mixed with a cargo in a dialysis bag, which is then exposed to a hypotonic buffer, again leading to the formation of pores through which the cargo can enter the RBCs [55]. Figure 3 shows a schematic illustrating optical cargo loading of RBCs, EGs, and nEGs.

It is interesting to note that in some cases, loading the RBCs with an optical cargo in a hypotonic buffer has been reported to preserve the biconcave disk shape and size of native RBCs. For example, Jiang et al. were able to load mouse RBCs with fluorescent silicon NPs hypotonically, resulting in constructs with the biconcave disk morphology ~6 μm in diameter [53]. Here, the hypotonic buffer was 0.5× PBS containing 2 mM ATP and 3 mM glutathione, and the mixture was incubated for 45 min at 4 °C. Jiang et al. showed that after the RBCs were resealed, the silicon NPs were visible inside the RBCs [53]. However, hypotonically loading the optical cargo does not always preserve the biconcave morphology of native RBCs, as can be seen by the constructs fabricated by Bustamante López and Meissner [56]. After using a hypotonic lysis buffer to load bovine erythrocytes with fluorescein isothiocyanate (FITC) glycylglycine conjugates, the resulting constructs were significantly smaller and had less hemoglobin compared to native erythrocytes. When analyzed using atomic force microscopy (AFM), the authors also found that the loaded constructs were more rounded and had a rougher surface compared to native erythrocytes. Here, the lysis buffer contained MgCl_2_, EDTA, phosphate buffer, and urea [56].

As demonstrated by the two previous examples, the buffer choice for hypotonic loading can vary, which may be why hypotonic loading does not always produce constructs that preserve native RBC morphology. A number of different buffers have been used for hypotonic dialysis [55], but there are no clear trends for hypotonic buffer choice when it comes to loading optical cargos. Another factor potentially affecting the morphology of the construct is the species source of blood. For instance, Jiang et al. used mouse blood for their constructs [53], while Bustamante López and Meissner used bovine blood [56]. Many constructs are fabricated using mouse blood. This includes the constructs engineered by Marvin et al., with RBC morphology preserved after using dialysis to load RBCs in a hypotonic PBS buffer with 6 mM glucose [57]. It is possible that differences between the RBCs, such as membrane composition, age, and size, could have an effect on how the cells react to hypotonic loading methods. It is important to note that for clinical translation, the constructs will ultimately need to be fabricated using human RBCs. It is known that there are a number of structural differences between mouse and human RBCs, such as size and membrane protein composition [58]. Further research is needed to determine whether hypotonic loading methods used on human RBCs will still preserve the native RBC morphology. More research is also needed on the effects of using different hypotonic buffers for the loading procedure, as well as the effect of the type of optical cargo being loaded.

Another important factor to consider with hypotonic treatment is the potential for phosphatidylserine (PS) exposure. PS is generally confined to the inner leaflet of erythrocytes [59], and its exposure leads to macrophage recognition and subsequent clearance of erythrocytes from circulation [60]. Wang et al. have shown that EGs prepared by incubating mouse RBCs in 5 mM NaH_2_PO_3_/Na_2_HPO_3_ for 5 min at 4 °C have significantly more PS exposure compared to native mouse RBCs [61]. Sun et al. showed similar PS exposure results for murine RBC-derived EGs, but showed that RBCs loaded with DOX via hypotonic dialysis did not show elevated PS exposure levels [62]. We have also shown elevated expression levels of PS on μEGs and nEGs following hypotonic treatment of bovine RBCs [63]. Therefore, the effect of hypotonic loading on PS exposure needs to be more thoroughly understood, particularly for carriers derived from human RBCs.

##### Other Methods for Interior Optical Cargo Loading

Electroporation is another method for cargo loading that involves the application of an electric field to the membranes, which results in the formation of pores through which materials can cross the membrane [64]. Bustamante Lopéz and Meissner were able to use electroporation to load bovine RBC membranes with glycylglycine-conjugated FITC [56]. Compared to loading using a hypotonic buffer, however, RBCs loaded via electroporation had a significantly lower loading efficiency [56].

Another loading method involves extrusion to load small organic molecules. In this case, the RBC membrane does not coat the cargo and is instead used as a nano-sized carrier. For example, we mixed EGs derived from bovine RBCs with ICG-BSA and extruded the solution, resulting in nano-sized constructs capable of being used for cancer cell imaging applications [65]. It is important to note that using extrusion to load organic molecules will result in a nano-sized construct. While these two examples demonstrate that there are potentially RBC interior loading methods that do not use a hypotonic buffer, hypotonic loading mechanisms are more common for loading optical cargos. More research is needed to determine whether these alternative loading methods offer a significant advantage over hypotonic loading methods. In addition, both of these examples were conducted with bovine RBCs. Mouse and human RBCs may respond differently to these loading techniques, indicating another area for further research.

##### Optical Cargo Embedding in the Lipid Bilayer

Some organic molecules are capable of directly interacting with RBC membranes, allowing them to be embedded into the lipid membrane of the RBC. For example, chlorin e6 (Ce6) is a lipophilic molecule capable of interacting with lipid membranes [66]. Gao et al. and Sun et al. have demonstrated that Ce6 can be embedded in RBC membranes by mixing [52,67]. Sun et al. used the Ce6-embedded RBC membrane as a coating for PB NPs [67], suggesting that embedding optical cargos can be combined with using RBC membranes as a substrate coating. Gao et al. demonstrated that they were able to combine the hypotonic dialysis loading of DOX into RBCs with Ce6 embedding in the RBC lipid bilayer [52]. In addition, Gao et al. were able to show that while DOX was loaded to the interior of the RBCs, Ce6 was localized to the membrane of the RBCs. Not only do these two studies demonstrate that optical cargos have the potential to be loaded into RBCs through simple mixing, but they also demonstrate that multiple loading methods can be used to fabricate a single construct. Another lipophilic dye, DiR, has been used for cell membrane labeling [68], and is another potential optical cargo that can be directly embedded in RBC membranes. Su et al. have demonstrated that DiR is capable of being embedded into the lipid membranes of nEGs, which are then used to coat paclitaxel-load polymeric NPs [32]. While this example of an optical cargo being embedded in the lipid bilayer was also ultimately used for substrate coating, it also suggests that lipophilic dyes can be embedded into the lipid membranes of EGs and nEGs. However, organic molecules are the only optical cargos that have been embedded into the lipid bilayer of RBC-derived constructs, so this loading method may have limited applications for other optical cargos, such as metallic NPs.

#### 2.2.2. Methods for Exterior Loading of an Optical Cargo

While RBC-derived constructs employed as carriers usually involve loading an optical cargo to the interior, or in some cases into the lipid membrane bilayer of the construct, there are some examples of the optical cargo being incorporated onto the surface. For example, some optical cargos are able to adsorb directly to the surface of RBCs. Delcea et al. were able to incorporate gold NPs into their constructs using adsorption. They suggest that the adsorption is possible due to interactions between the positively charged NPs and the negatively charged glycocalyx on the RBC membrane [18].

It has also been suggested that organic molecules are capable of adsorbing to RBCs; however, the results have not been as promising. Sun et al. report that they were able to adsorb ICG to the surface of RBCs, but that the ICG was easily washed away in serum-supplemented media [62]. Flower and Kling similarly showed that while ICG is capable of adsorbing to RBCs, the amount of ICG that could be loaded was almost half the amount that could be loaded into RBCs hypotonically [69]. While there is potential for optical cargos to adsorb directly to RBCs, more research needs to be done to determine the types of optical cargos it is appropriate for, as well as determining how the adsorption is accomplished for those appropriate optical cargos.

Another method for attaching an optical cargo to the surface of RBC-derived constructs is functionalization. Here, a lipid linker is used to attach the optical cargo to the surface of the construct. A common lipid linker is 1,2-distearoly-sn-glycero-3-phosphoethanolamine-N-[χ(polyethylene glycol)] (DSPE-PEG-χ) where χ can be a number of functional groups. DSPE is able to insert itself into the lipid bilayer of the RBC membranes, similar to the lipid-insertion methods outlined by Fang et al. [70], resulting in the desired molecule being attached to the surface of the RBC membrane. Wang et al. incubated RBCs with DSPE-PEG-biotin, which enabled them to attach various avidin-functionalized UCNPs to the RBCs [71,72]. This method takes advantage of the strong and highly specific interaction between biotin and avidin [73], resulting in constructs that preserve the biconcave morphology of native RBCs [71,72]. Other groups have used other lipid linkers to biotinylate RBCs. For example, Tang et al. used biotin-X-NHS, which was able to attach to neutravidin, and in turn able to bind to their biotinylated ferritin nano-capsules loaded with the optical cargo ZnF_16_Pc [74]. Wang et al. used sulfo-NHS-LC-biotin to biotinylate RBCs, which could then bind to Ce6-coated iron oxide NPs that were conjugated to avidin [61]. While there are only a few examples of functionalizing RBCs with an optical cargo, there are a number of groups looking at functionalizing RBC-derived constructs with other moieties for active targeting, as will be discussed below.

## 3. Potential Clinical Applications

### 3.1. Imaging Applications

#### 3.1.1. Fluorescence Imaging

RBC-derived constructs have been developed for a number of light-based applications. How the optical cargo interacts with light will determine which light-based applications the RBC-derived construct is suitable for. If light excitation results in the optical cargo generating fluorescence emission, it can be used for various imaging applications. For example, Guo et al. developed constructs made from hydrophobic QDs coated in membrane lipids extracted from RBCs [16]. The constructs are able to fuse with the cell membrane, allowing the QDs to be embedded in the membrane for imaging applications as well as tracking cell movement. Our group was the first to demonstrate the effectiveness of nano-sized particles derived from RBCs and containing ICG in NIR fluorescence imaging of mammalian cells [22].

In addition to tracking cell movement using fluorescence imaging, RBC-derived constructs can be designed for cancer imaging. For example, Li et al. prepared NPs from gold nanoclusters and pea protein isolate that were coated with RBC membranes, resulting in nano-constructs suitable for NIR fluorescence imaging in tumor bearing mice [75]. The RBC membrane coating resulted in increased tumor accumulation of the NPs compared to bare NPs. Another imaging construct developed for cancer imaging involves loading nEGs with ICG-BSA for enhanced fluorescence emission as compared to emission of ICG alone [65]. We found that SKOV3 ovarian cancer cells had a higher uptake efficiency of these constructs compared to free ICG-BSA.

ICG has also been used in constructs developed for vascular fluorescence imaging. In 2008 Flower et al. developed the first light-based RBC-derived construct when they hypotonically loaded RBCs with ICG. These constructs have been used to monitor blood flow in the retinal capillaries and choriocapillaris in monkeys [50], and characterize microvascular vasomotion in the human eye and skin [69]. These did not involve sonication/extrusion methods, and were prepared under sterile conditions [69]. The only other human study to date, conducted by Wang et al., focused on using the constructs developed by Flower et al. to automate the process of estimating the velocity of erythrocytes in retinal arterioles and venules [76]. We and our collaborators have demonstrated the real-time circulation dynamics of micro- and nano-sized RBC-derived particles containing ICG by NIR fluorescence imaging of mice cutaneous microvasculature [63]. In collaboration with others, we have also used nano-sized RBC-derived particles containing ICG for both fluorescence and photoacoustic imaging of atherosclerotic lesions and occlusions due to myocardial infarction in mice [77].

In addition to vasculature imaging, organic chromophores combined with RBC-based platforms can also be used for blood analyte sensing. Meissner’s group has developed constructs referred to as erythrosensors for monitoring blood plasma parameters [21,51]. These constructs are loaded with glycylglycine-FITC, a pH-sensitive dye. They found that the erythrosensors were able to respond to changes in extracellular pH as shown by changes in the measured fluorescence intensity of the constructs [51]. Theoretically, these erythrosensors could circulate throughout the body over time and monitor the blood pH of a patient any time the particles are exposed to an excitation light source. While this is not a direct imaging application, they also show that the resulting constructs are still visible in epifluorescence micrographs, and they rely on fluorescence emission for their application. In theory, other dyes that are sensitive to specific blood analyte levels, such as glucose levels, could also be loaded into RBCs and used as blood analyte sensors over time [51].

#### 3.1.2. Upconversion Luminescence Imaging

If an optical cargo is able to take low-energy photons and convert them into higher-energy photons, it can be used for upconversion luminescence imaging (UCL) [29,78]. UCNPs are a promising optical cargo due to their low photobleaching and the potential ability to overcome autofluorescence [78]. Multiple groups have investigated combining UCNPs with RBC-based delivery constructs, particularly for cancer imaging. For example, Li et al. prepared NaGdF_4_:Yb, Tm UCNPs that were then coated with RBC membranes [42]. The resulting constructs showed increased tumor uptake compared to bare UCNPS, and could be used for UCL, as well as magnetic resonance imaging (MRI) and positron emission tomography (PET) imaging [42]. Similarly, Rao et al. prepared β-NaYF_4_:Er^3+^, Yb^3+^ UCNPs coated with RBC membranes and reported increased tumor uptake of the resulting constructs compared to bare UCNPs [36]. These examples show that combining UCNPs with RBC-based constructs have strong potential for use in cancer imaging and detection. When combined with other treatment modalities, as will be discussed below, UCNPs represent a promising new optical cargo.

### 3.2. Phototherapy

#### 3.2.1. Photothermal Therapy

When light absorption of an optical cargo results in a temperature increase, the optical cargo can be used for PTT. We reported the first demonstration of nano-sized particles derived from RBCs and containing ICG for photothermal destruction of mammalian cells [22].

Heat generated during PTT has potential applications in the treatment of thrombi [79], port wine stains (PWSs) [63], and cancers [80]. For example, Li et al. developed gold nanorods coated with RBC membranes for PTT of human lung carcinoma cells in vitro after 808 nm laser irradiation [81]. Piao et al. also used a gold-based optical cargo coated with RBC membranes for PTT [82].

Magnetic materials, especially those made from iron oxide, are another promising PTT agent [83] commonly used in RBC-derived constructs. It is important to note that since iron oxide NPs are approved for use as MRI contrast agents in humans [83], many of these constructs are developed for PTT in combination with MRI. For instance, Fe_3_O_4_ magnetic NPs and nanoclusters coated with RBC membranes have been used for MRI-guided PTT of tumors in mice [24,84].

While light-based RBC-derived constructs designed for PTT are mostly being investigated for cancer applications, there are other potential uses that are starting to be explored. We have demonstrated photothermal destruction of mice cutaneous microvasculature using pulsed 755 nm laser irradiation in conjunction with ICG-containing microparticles [63]. This approach may become applicable for laser treatment of hypervascular cutaneous malformations, known as PWSs.

#### 3.2.2. Photodynamic Therapy

Optical cargos can be used for PDT when light absorption generates reactive oxygen species (ROS) [80]. Common PDT agents include tetrapyrrole organic molecules as well as various NPs including QDs and UCNPs [85]. A vast majority of light-based RBC-derived constructs fabricated for PDT are developed for cancer applications, which has its own specific challenges researchers must address when designing their constructs. For instance, many cancers result in hypoxic tumors [86]. Since one of the main mechanisms of PDT is dependent on converting O_2_ into ROS, the lack of oxygen in tumors is a challenge for effective PDT [87]. In order to enhance PDT for cancer treatment, a number of groups have investigated incorporating oxygen delivery in addition to optical cargos in RBC-derived platforms. Two common methods are to use hemoglobin or perfluorocarbons (PFC) as oxygen carriers [87]. For example, Liu et al. loaded RBC membranes with complexes made from hemoglobin as an oxygen carrier, polydopamine to protect the hemoglobin from oxidation, and MB as an optical cargo [88]. Here, laser irradiation at 660 nm excites the MB, which has shown great promise as a PDT agent [89], while the hemoglobin and polydopamine ensure there are adequate oxygen levels at the treatment site [88]. Another example is the construct fabricated by Tang et al., where RBCs are functionalized with ferritin nanocages loaded with the photosensitizer ZnF_16_Pc [74]. The photosensitizer is excited by 671 nm laser irradiation and then the hemoglobin inside the RBCs provides an oxygen source. These constructs demonstrated efficient PDT in an in vivo hypoxic tumor model [74].

In addition to usually being hypoxic, many tumors produce large amounts of hydrogen peroxide (H_2_O_2_), which may play a role in tumor metastasis and cell proliferation [90,91]. In normal cells, catalase is present to decompose H_2_O_2_ into O_2_ and water, but many cancer cells also appear to have a catalase deficiency [91,92]. RBCs naturally contain catalase, and Du et al. have shown that RBC-derived particles can be developed for catalase delivery in conjunction with a sonosensitizer to enhance sonodynamic therapy in a hypoxic environment [93]. Therefore, there is potential for combining catalase and an optical cargo in an RBC-derived platform for enhanced PDT in a hypoxic tumor environment. Researchers have also been able to design optical cargos that take advantage of the high H_2_O_2_ levels in tumors. Zhao et al. designed a metal–organic framework (MOF) consisting of FeTCPP/Fe_2_O_3_ capable of decomposing H_2_O_2_ and promoting the formation of singlet oxygen as well as hydroxyl radicals [94]. When these MOFs are coated with RBC membranes they exhibit strong in vivo PDT after 660 nm laser irradiation. This example demonstrates that by carefully designing the optical cargo, RBC-based constructs can overcome the unique challenges presented by the tumor environment.

#### 3.2.3. Combined Phototherapies

In order to increase therapeutic efficacy, some researchers have designed RBC-based constructs that are capable of both PTT and PDT, particularly for cancer applications. One method to enable both PTT and PDT capabilities is to use multiple optical cargos. For example, Sun et al. embedded Ce6 in RBC membranes which were used to coat PB NPs [67]. Laser irradiation at 660 nm resulted in Ce6-mediated PDT as well as membrane rupture, while laser irradiation at 808 nm led to PTT from the PB NPs. In vivo results show these constructs, in conjunction with dual laser irradiation, have excellent tumor growth inhibition abilities [67]. Another example is the construct designed by Li and Zhang, where gold nanorods and TiO_2_ NPs were incorporated in RBC membranes [95]. In combination with both UV and NIR light irradiation, the constructs were used for PTT as well as PDT, and showed promising results for cancer cell destruction in vitro.

While incorporating multiple optical cargos does allow for these constructs to be used for both PTT and PDT, it is important to note that many optical cargos are capable of being used for more than one therapeutic effect. For example, Liu et al. have shown that Cu_2-x_Se NPs coated with RBC membranes can be used for both PDT and PTT in the treatment of tumors after laser irradiation at 1064 nm [20]. We have demonstrated that ICG within RBC-derived nanoparticles can mediate both PTT and PDT effects [96].

By carefully selecting the optical cargo used, researchers can achieve multifunctionality of RBC-derived constructs without the need for multiple optical cargos, and limit the need for more than one laser. Simplifying the designed platforms in this way may ease the process for clinical translation, while still allowing for PTT and PDT to potentially be performed simultaneously.

### 3.3. Drug Delivery

While a majority of light-based RBC-derived constructs are designed for imaging and phototherapy applications, there is a small subset of constructs that are being designed specifically for light-based drug delivery. Hughes et al. conjugated various bioactives including colchicine, paclitaxel, and methotrexate, to cobalamin [97]. These compounds were hypotonically loaded into RBCs, and after being illuminated at 525 nm the Co-C bond between the bioactive and the cobalamin was cleaved, resulting in the drug being released from the interior of the erythrocytes. Hughes et al. also demonstrated that the wavelength for the cleavage of the bonds between the bioactives and cobalamin could be tuned by conjugating fluorophores, such as Cy5, to the compounds [97]. This finding suggests that the compounds could be tailored for different light sources; however, most light-based RBC-derived constructs that are engineered for drug delivery are also designed for other applications that will be discussed below.

### 3.4. Combining Applications

#### 3.4.1. Light-Based Theranostics for Optical Imaging and Phototherapy

**Figure 4:**
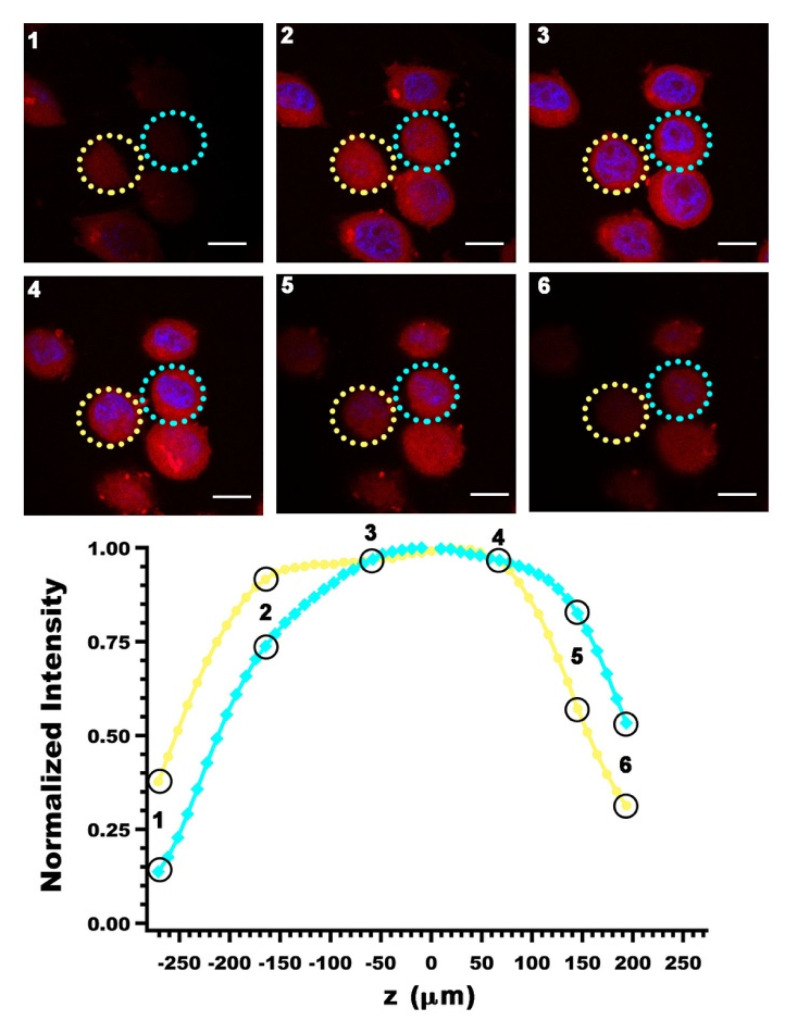
Z-stacks of SKBR3 breast cancer cells images obtained by laser scanning confocal fluorescence microscopy. Images were obtained three hours after incubating the cells with ICG-nEGs at 37 °C, and are falsely colored. Blue channel: DAPI staining of the nucleus; red channel: NIR emission from ICG-nEGs internalized by the cells. Values of normalized fluorescence emission intensity associated with the circled region on each stack are plotted as a function of relative depth of the corresponding stack. Scale bar = 10 µm in all panels.

**Figure 5:**
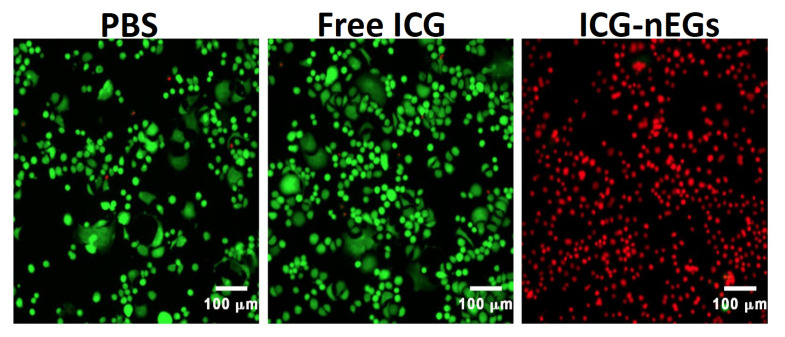
Fluorescence images of SKBR3 breast cancer cells following 808 nm laser irradiation at irradiance of 680 mW/cm^2^ for 15 min. Cells were incubated with PBS (negative control), free ICG (positive control), or ICG-nEGs for three hours at 37 °C, and then washed three times before laser irradiation. Live cells are identified by Calcein acetoxymethyl staining, and falsely-colored in green. Dead cells are identified by ethidium homodimer-1 (EthD-1), and falsely-colored in red.

While there are a number of light-based RBC-derived constructs that are formulated to be used for only one application (i.e., imaging, drug delivery, or phototherapy), it is common for these constructs to be designed for multiple applications. One of the more common application combinations is to incorporate both imaging and phototherapeutic capabilities. Similar to how multiple optical cargos can be incorporated into a construct in order to allow the final product to be used for both PTT and PDT, using multiple optical cargos can also achieve multifunctionality with imaging and phototherapy. For example, Wang et al. have developed RBC-derived theranostic probes that involve ICG-BSA, RB, and UCNPs [72]. The ICG-BSA is hypotonically loaded into RBCs and excited by 808 nm laser irradiation for imaging and heat generation that causes the release of oxygen from the RBCs. The UCNPs are then excited by 980 nm laser irradiation, resulting in 550 nm light that excites the RB for PDT using the released oxygen as a source for generating ROS. We present an illustrative example of our own work in using ICG-nEGs for NIR fluorescence imaging (Figure 4), and laser-mediated killing of SKBR3 breast cancer cells in vitro (Figure 5).

Many optical cargos are capable of being used for more than one therapeutic effect. For instance, we have shown that NPs fabricated from nEGs hypotonically loaded with just ICG were able to be used for imaging as well as PTT and PDT for tumor treatment [96]. These constructs are capable of generating ROS. After treatment in vivo, extracted tumors showed higher levels of photo-induced activation of Capase-3 [96], which has been linked to PDT-induced apoptosis [98,99,100,101]. It is interesting to note that in this case, the PDT was performed in a hypoxic environment without extra catalase or oxygen delivery. This could be due to the combined effects of PTT with PDT. There have been recent investigations towards using PTT to increase tumor blood flow and O_2_ levels, both of which would increase the efficacy of PDT in hypoxic environments [87].

**Figure 6:**
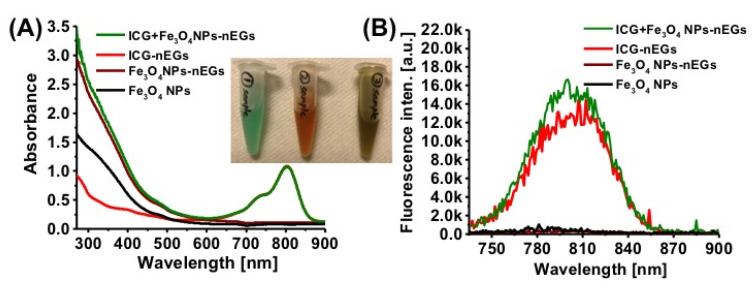
(**A**) Absorption and (**B**) fluorescence emission spectra of nEGs loaded with ICG and Fe_3_O_4_ NPs (ICG + Fe_3_O_4_ NPs-nEGs). For comparison, spectra for Fe_3_O_4_ NPs, ICG-nEGs, and nEGs loaded with Fe_3_O_4_ NPs (Fe_3_O_4_ NPs-nEGs) are also shown. Diameters of Fe_3_O_4_ NPs were in the range of ~ 5–10 nm. nEGs were formed by extrusion of EGs, and then loaded with ICG and Fe_3_O_4_ NPs. Concentrations of ICG and Fe_3_O_4_ NPs in the hypotonic loading buffer were 20 µM and 1 mg/mL, respectively. The inset on panel (**A**) shows, from left to right, photographs of ICG-nEGs, Fe_3_O_4_ NPs-nEGs, and ICG + Fe_3_O_4_ NPs-nEGs suspended in isotonic PBS. Absorption > 600 nm is due to ICG. Fluorescence emission was recorded in response to photoexcitation at 720 nm. Presence of Fe_3_O_4_ NPs in ICG + Fe_3_O_4_ NPs-nEGs had minimal effect on absorption and fluorescence emission characteristics of the particles as compared to those for ICG-nEGs.

In addition to having an optical cargo, RBC-derived constructs can be co-loaded by non-optical agents to provide a multi-functional platform. For example, our group has loaded nEGs with ICG and Fe_3_O_4_ magnetic NPs (Figure 6). These constructs can potentially be used for dual MRI and fluorescence imaging as well as PTT. Wang et al. designed Fe_3_O_4_ magnetic nanoclusters that were loaded with cypate before being coated with RBC membranes [40]. Here, 808 nm laser irradiation of the cypate is used for in vivo fluorescence imaging and PTT, while the Fe_3_O_4_ magnetic nanoclusters allow the constructs to be used for MRI. There are a large number of potential optical cargos that can be used to give RBC-based constructs both imaging and phototherapeutic abilities. By combining them with other cargos researchers can continue to develop more constructs that can be tailored to specific disease states.

#### 3.4.2. Imaging and Drug Delivery

Another combination of applications is imaging and drug delivery. In some cases, the optical cargo is used not only as an imaging agent, but also as a catalyst for drug release. For instance, Marvin et al. designed a light-response vitamin drug conjugate composed of Cy5, vitamin B12, and the chemotherapeutic drug taxane [57]. Once the conjugates are loaded into RBCs, the membrane impermeable B12 ensures the drug remains inside the carrier until it has reached its target. Then, Cy5 is able to be excited by 650 nm wavelength light, cleaving the bond between taxane and B12, resulting in taxane being able to leave the RBC carrier [57]. Not only were the constructs able to be used for site-specific drug release, but they were also used for imaging in vivo.

Another example of an RBC-derived construct that combines drug delivery and imaging capabilities is the construct fabricated by Liu et al. Zinc gallogermanate nanostructures were coated with mesoporous silica and then loaded with DOX before being coated with RBC membranes [102]. The constructs produced an NIR signal due to the zinc gallogermanate nanostructures that was visible in vivo for up to 48 h, and the anti-tumor activity of the constructs outperformed a control injection of just DOX.

While both of the previous examples are specifically for cancer imaging and treatment, there is a clear potential for therapeutic imaging and drug delivery to be applied to vascular targets. As a prospective RBC-based thrombus therapy, we have developed nano-sized constructs of ICG loaded nEGs functionalized with tissue plasminogen activator (tPA) [103]. The tPA allows the constructs to target the fibrin in the clot, while the ICG loaded into the nEGs allows for NIR fluorescence imaging of the clot. We showed that these constructs were able to induce lysis of blood clot models [103].

#### 3.4.3. Phototherapy and Drug Delivery

Combining PTT and/or PDT with drug delivery has been used for light-based applications using RBC-derived constructs. For example, Pei et al. developed RBC membrane-camouflaged NPs with a polymeric core containing dimers of the chemotherapy drug paclitaxel as well as 5,10,15,20-tetraphenylchlorin (TPC) as an optical cargo for PDT [104]. On the other hand, Wang et al. recently reported the design of RBC membrane-coated BSA nano-constructs loaded with ICG for PTT, and gambogic acid as a chemotherapeutic [37]. With a large number of potential optical cargos and chemotherapeutics available, the possible combinations could result in many different RBC-derived particle formulations. By continuing to investigate different optical cargo and drug combinations, it is possible that multiple therapies will be developed and can be tailored to the treatment of specific cancers. However, the most common drug investigated for use in RBC-derived constructs is DOX. DOX is currently used to treat various cancers including breast, ovarian, bladder, and lung cancers [105].

Chen et al. fabricated DOX-based constructs by loading hollow mesoporous PB NPs with DOX, and coated them with RBC membranes. They demonstrated strong PTT and anti-tumor effects in vivo after NIR laser irradiation [28]. Another DOX example designed by Hui et al. involves loading DOX and ICG in upper critical solution temperature (UCST) polymer micelles that are then coated with RBC membranes [106]. These particles were also capable of efficient PTT, and their cytotoxicity in vitro was increased after NIR laser irradiation.

The previous examples combine drug delivery with either PTT or PDT, but not both. However, it is possible to design constructs capable of drug delivery, PTT, and PDT simultaneously. This can be achieved by using an optical cargo capable of both PTT and PDT, such as ICG. For instance, Luo et al. loaded graphene oxide QDs with ICG and DOX which were then loaded into nEGs [107]. The constructs showed strong PTT and PDT effects in vitro, and in combination with the drug delivery and 808 nm laser irradiation had an impressive anti-tumor effect in vivo. Another example that incorporates ICG is the previously mentioned NPs made by Liu et al. RBC membranes are used to coat nanocomplexes containing BSA, ICG, and DACHPt as a chemotherapeutic agent [48]. The particles were capable of in vitro hyperthermia and ROS generation after NIR laser irradiation, and demonstrated efficient anti-tumor and anti-metastatic abilities in vivo. The authors note that the drug release was enhanced by the NIR laser irradiation, which suggests that the drug release mechanism is mediated by light activation [48].

All of these examples illustrate the powerful cancer applications that use light-based RBC-derived constructs for combined phototherapy and drug delivery. However, there are also examples of phototherapy and drug delivery constructs being used for other potential clinical applications. Shao et al. have developed Janus-type polymeric micromotors coated with RBC membranes capable of being directed to thrombus sites using NIR light irradiation [108]. Here, the core of the construct is a capsule made of heparin and chitosan that is partially coated in gold before encapsulation in nEGs. NIR laser irradiation of the gold coating led to a temperature gradient around the construct, enabling its directed motion using a thermophoretic effect [108]. In addition to using NIR light to induce directed movement of the constructs, the NIR light was able to induce photothermal destruction of thrombus models. The generated heat also mediated the release of heparin from the constructs to aid in thrombolysis. This example demonstrates that in addition to having great promise in the realm of cancer treatments, light-based RBC-derived constructs can be designed for phototherapy and drug delivery for vascular applications.

#### 3.4.4. Imaging, Phototherapy, and Drug Delivery

As previously mentioned, a common method for increasing the number of applications a construct can be used for includes using optical cargos capable of more than one application. By carefully choosing an optical cargo that can be used for imaging and phototherapy and combining it with a drug, some light-based RBC-derived constructs can be used for imaging, phototherapy, and drug delivery simultaneously. Here, it is very common to choose an organic molecule as the optical cargo. As previously mentioned, Ye et al. engineered constructs where the chemo-drug HCPT and ICG co-assembled NPs are coated with RBC membranes. Here, ICG is used for fluorescent imaging after NIR laser irradiation, as well as PTT. The generated heat disrupts the construct’s membrane, causing the release of the HCPT [27]. Similarly, Su et al. developed mesoporous silica NPs co-loaded with DOX and the NIR dye Ce6 coated with RBC membranes [109]. After laser irradiation, Ce6 was not only able to serve as a fluorescent probe, but also resulted in the generation of ROS. The authors hypothesized that the ROS generation could not only destroy tumor cell membranes, but the EG membranes as well, leading to the release of DOX from the construct.

PB NPs are another common optical cargo for constructs that can be used for imaging, phototherapy, and drug delivery. Liu et al. have demonstrated that hollow mesoporous PB NPs can be loaded with the chemotherapeutic gamabufotalin before being encapsulated in RBC membranes [110]. After 808 nm laser irradiation the constructs were capable of being used for NIR fluorescence imaging as well as displaying effective anti-tumor activity from the PTT and drug delivery [110].

It is important to note that since many optical cargos are capable of being used for more than one light-based application, it is possible that some constructs are capable of being used for imaging, phototherapy, and drug delivery, despite only being designed for one or two of those applications. For instance, since ICG has been used for imaging, PTT, and PDT [111], constructs fabricated with ICG can potentially be used for all three applications even if they were only initially designed for one. Therefore, many light-based RBC-derived constructs are inherently endowed with multifunctionality, representing a promising treatment platform that has been particularly studied for cancer applications.

### 3.5. Specific Considerations for Targeting Cancer Cells

PDT and PTT are two of the more common light-based treatment modalities involving RBC-derived particles. Both PDT and PTT are being heavily investigated for their use in the treatment of cancer [112], so it follows that a large number of light-based RBC-derived platforms are designed specifically for applications in the treatment of various cancers. There is a recent review by Sun et al. that covers a number of different RBC-derived formulations explicitly fabricated for the treatment of cancer [13], with a focus on platforms that involve the various functionalization methods for conferring NPs with targeting capabilities. In addition, RBC-derived constructs have also been extensively reviewed for their drug delivery abilities [4,6,7,8,10], including their utility in drug delivery for anti-tumor applications [113].

In order for RBC-derived constructs to have any anti-cancer efficacy they need to be able to reach their target efficiently. Targeting various types of cancer cells can be accomplished using multiple techniques during the design phase for engineering of RBC-derived particles. For instance, a majority of these formulations are on the nano-scale, meaning they are capable of passively targeting cancer cells via the enhanced permeability and retention (EPR) effect [114]. Tumor vasculature is abnormal and hyperpermeable [115], which enables NPs to more readily accumulate in tumors compared to healthy tissues. While many constructs rely only on the EPR effect in order to ensure cancer targeting, the EPR effect has been shown to be heterogeneous in humans [116]. Researchers have therefore started to employ various functionalization methods to bestow their constructs with more active targeting capabilities, bypassing the need for passive targeting via the EPR effect. Based on our review of the literature, a common method for functionalizing RBC membranes involves DSPE-PEG-χ lipid insertion. As previously mentioned, DSPE is able to insert itself into the lipid bilayer of the RBC membranes, resulting in the desired functional group, χ, being attached to the surface of the RBC membranes. Common functional groups include folate or folic acid (FA), which is able to target folate receptors overexpressed on a number of different cancer cells [117,118]; as well as various formulations of RGD peptides, which are able to selectively bind to α_V_β_3_ integrins [119], which are also expressed on some tumor cells [120]. Examples of light-based RBC-derived constructs that are functionalized via DSPE lipid insertion are listed in Table 2.

**Figure 7:**
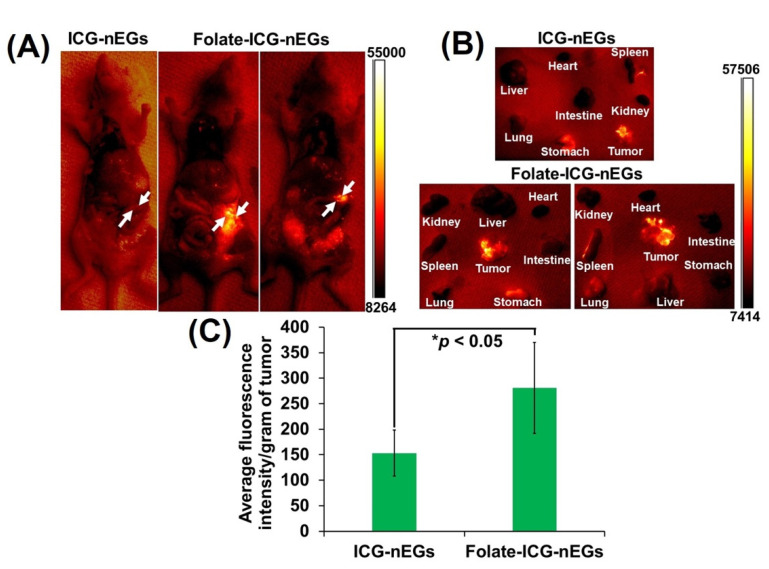
(**A**) Open-abdomen whole-body NIR fluorescence images of immunodeficient mice inoculated with intraperitoneal SKOV ovarian cancer cells at 24 h post-intravenous administration of ICG-nEGs, or folate-functionalized ICG-nEGs (folate-ICG-nEGs). Images are falsely colors with the emission intensity scale shown on the right side. White arrows point to the tumors. (**B**) Corresponding images of the extracted organs and tumors from each mouse. (**C**) Average fluorescence emission intensity normalized to weight of each tumor, and subsequently averaged among the number of homogenized tumors (*n* = 3 tumors from three different animals). Asterisk indicates a statistically significant difference (*p* < 0.05). For imaging of the whole body, and the extracted organs and tumors, photoexcitation wavelength was 710 ± 25 nm, and emission > 785 nm was recorded. Homogenized tumors were photoexcited at 720 nm, and emission in 735–900 nm band was recorded.

While relatively few functionalized RBC-derived constructs have been developed for light-based imaging and therapy, almost all of them have been designed for cancer applications. For example, Luo et al. designed RBC-derived ICG and DOX-containing NPs for the chemo-PPT of cervical cancer [107]. After functionalizing the NPs with FA, the researchers observed a significant improvement in the tumor accumulation of their constructs. We have functionalized ICG-nEGs with folate, and used them for NIR fluorescence imaging of intraperitoneal ovarian tumors in mice. Our results indicated that there was greater accumulation of folate-functionalized ICG-nEGs (folate-ICG-nEGs) in tumors as compared to ICG-nEGs (Figure 7).

Other groups have also been able to use functionalization to improve the targeting ability of micro-sized constructs that would not be able to rely on the EPR effect for tumor accumulation. Wang et al. functionalized RBCs with UCNPs coated in RGD peptides and demonstrated that the RGD peptides supplied their constructs with tumor targeting abilities in vivo [72].

In addition to functionalization, other targeting methods include the application of various stimuli, including magnetic and acoustic fields. For instance, Gao et al. showed that RBC membrane-wrapped particles containing Fe_3_O_4_ NPs and ICG could be propelled using an acoustic field, and that the direction of these particles could be controlled using a magnetic field for the targeted delivery of ICG for PDT [49]. Other groups have shown that loading RBC membrane-derived particles with magnetic mesoporous silica NPs [125], Fe_3_O_4_@Cu_2-x_S NPs [126], and Fe_3_O_4_ NPs [23] increased the tumor uptake of the particles when combined with the application of a magnetic field.

Another recently developed targeting method is combining the RBC membranes with the membranes of other cells to create hybrid membrane coatings. Liu et al. combined RBC membranes and platelet membranes and showed that constructs derived from those hybrid membranes had more tumor accumulation compared to constructs fabricated from just RBC membranes or platelet membranes [127]. The platelet membranes give the constructs the ability to target microthrombi, which are created when NIR light is applied to the tumor, resulting in the enhanced tumor accumulation. Jiang et al., has also had success in enhancing tumor targeting by combining RBC membranes with MCF-7 cancer membranes to coat melanin NPs [128]. They showed that the membrane of cancer cells could be used for the homotypic targeting of MCF-7 cancer cells and showed enhanced tumor PTT when the RBC:MCF-7 membrane ratio was 1:1. Wang et al. also used cancer cell membranes to make hybrid membranes when they fused RBC membranes with melanoma B16-F10 cell membranes to coat copper sulfide (CuS) NPs [19]. They reported that the combination of the two cell membranes allowed for greater tumor accumulation of the CuS NPs compared to NPs coated with just one type of membrane. Overall, there are a number of different promising methods for endowing RBC-derived light-based constructs with the ability to target cancer cells. Since the field is still fairly young more targeting methods will undoubtably arise as researchers continue to push the boundaries of the field.

## 4. Trends in Biocompatibility and Toxicology

Overall, the in vitro results for studies with RBC-derived constructs that utilize light have been very promising. However, in vivo results will ultimately be more important for clinical translation and will be the focus of our discussion. There are a number of different experiments that researchers have been conducting in order to determine not only the efficacy of RBC-derived constructs, but also their biocompatibility. For instance, Wan et al. reported that after a series of injections of their RBC-derived constructs, there was no significant change in the mouse body weight, and there was no visible damage in the mouse organs as shown by histology [129]. Monitoring the mouse body weight and examining the organ histology are only two of the methods researchers use to determine the biocompatibility of drug delivery vehicles. Hematological profiling, such as white and red blood cell counts, mean corpuscular volume, hemoglobin, hematocrit, and platelet counts, is another method. We recently demonstrated that injection of micro- and nano-sized RBC-derived constructs did not result in any of the mentioned blood parameters being outside the normal range [46]. In addition, we demonstrated that RBC-derived particles had minimal effects on hepatic (alanine aminotransferase (ALT), aspartate aminotransferase (AST), and alkaline phosphatase (ALP)) and renal (urea nitrogen and creatinine) function assays.

While many of these tests are conducted in healthy mice, they can also be done in disease models, such as cancer, to demonstrate the effectiveness of the RBC-derived constructs for their designed application. For instance, Liu et al. showed that tumor bearing mice showed no organ damage after injections of their RBC-derived constructs with and without laser irradiation, but also showed that the damage to tumor tissue increased when the particles were combined with laser irradiation, as shown by histology [20]. Another example is the work done by Pei et al., where they showed that in tumor bearing mice, their RBC-derived constructs combined with laser irradiation led to no significant changes in mouse body weight, as well as no damage to organs, and without change in ALT, AST, uric acid, urea, and creatinine serum levels [104].

A majority of the in vivo results have focused on the biodistribution of the RBC-derived particles, and signs of toxicity. However, there have been few studies to assess the immunogenicity of RBC-derived particles. One example is the work done by He et al., where they evaluated the serum levels of cytokines interleukin (IL)-6, IL-12, and tumor necrosis factor (TNF)-α between 24 and 72 h after injection of RBC-derived particles. They reported that the RBC coated constructs did not induce elevated levels of the investigated cytokines compared to a control injection at any of the time points [26]. Sun et al. also showed that injections of their RBC-derived constructs did not induce elevated blood serum levels of TNF-α for up to 7 days post-injection compared to a control [67].

However, these studies did not examine the immediate immune responses, nor the effects of multiple injections of RBC-derived platforms. Rao et al. assessed the IgM and IgG levels in blood samples of mice that had received two injections of their NPs, and showed that there were no differences in the IgM and IgG levels compared to mice that had received one injection of the NPs, suggesting that the adaptive immune system had not been activated [130]. We have also recently investigated the acute innate immune response to intravenous administration of nano- and micro-sized particles derived from mice RBCs and containing ICG in healthy mice. We found that the nano-sized particles, in general, were associated with production of higher levels of the cytokines IL-6, TNF-α, and monocyte chemoattractant protein (MCP)-1 as compared to those produced in response to micro-sized particles, but lower than the levels induced by the immunogenic agent, lipopolysaccharide, within 6 h of injection [131]. We also showed that a second injection of these particles did not induce significantly elevated levels of these cytokines, which further suggests that RBC-derived particles do not activate the adaptive immune system. Therefore, there is a need for more research into the immunogenicity of RBC-derived particles, particularly when looking at the effect of multiple injections of the particles.

## 5. Outlook and Future Directions

Combining RBC delivery systems with light-based treatment modalities is an exciting field that is still being developed. Constructs are able to be designed in a number of different ways, allowing for them to be tailored to specific targets for seemingly endless applications. We have broadly divided light-based RBC-derived constructs into two main categories: constructs that use an RBC membrane as a coating, and constructs that use the RBC, EG, or nEG as a carrier. Deriving the constructs from RBCs endows their cargos with capabilities for delayed recognition and removal by the immune system, as well as the potential for increased circulation time. In addition, numerous methods have been employed to give RBC-derived constructs targeting capabilities. These methods include functionalizing the constructs with a targeting moiety; using cargos that can have their motion directed using light, magnetic fields, or acoustic fields; and combing RBC membranes with other cell membranes capable of specific interactions with the target.

Many of the studies discussed above have demonstrated that RBC-derived constructs are effective for light-based theranostics, particularly for cancer imaging, phototherapy, and drug delivery. However, we have also highlighted the potential for light-based RBC-derived constructs to be used in vascular applications as well. In addition, a majority of the studies suggest that these constructs have excellent biocompatibility as well as low toxicity. However, the field is still fairly young, and before many of these constructs can be used in a clinical setting, there is still a large amount of work to be done. For example, researchers are just beginning to probe the potential immune interactions of these constructs. While results are promising, understanding the potential immune response to any of these constructs is necessary before clinical translation can be realized.

**Figure 8:**
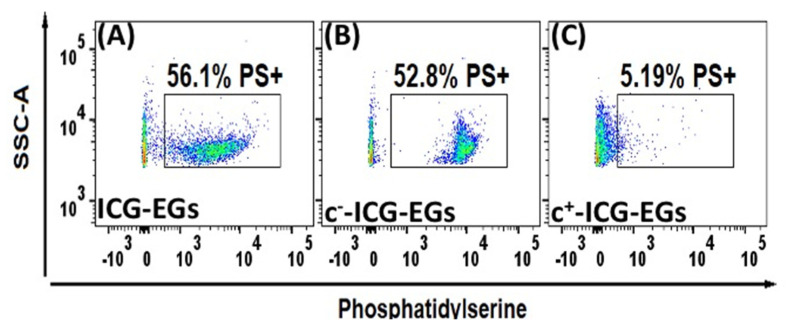
Quantification of PS on the outer leaflet of (**A**) ICG-EGs (negative control), (**B**) cholesterol-depleted ICG-EGs (c^-^-ICG-EGs) (positive control), and (**C**) cholesterol-enriched ICG-EGs (c^+^-ICG-EGs). Particles were fabricated using human RBCs, and loaded with ICG in hypotonic buffer. We show representative dot plots of side scattering vs. fluorescence emission of AlexaFluor 488-labeled annexin V, which targets surface-exposed PS. Boxed regions correspond to PS-positive particles. The indicated percentages refer to the fraction of the particles in the sample population that are PS-positive, and represent the average of four measurements on each sample.

Other important considerations in development of RBC-derived constructs for clinical translation relate to biochemical and mechanical integrity of the particles. In particular, during the fabrication process, flip-flopping of PS from the inner to the outer leaflet of the membrane bilayer can subsequently trigger recognition and phagocytosis by macrophages. PS surface exposure is also considered as an “eat-me” signal associated with removal of senescent RBCs by spleen macrophages. Using flow cytometry, we have determined that majority of ICG-EGs (~56% of particles) were PS-positive (Figure 8). One approach employed by our group to minimize flip-flopping of PS is to enrich the membrane of the RBC-derived particles with cholesterol. Our results indicate that this method is effective in retaining the PS within the inner leaflet of the membrane, and can reduce the fraction of PS-positive ICG-EGs to approximately 5% (Figure 8).

Normal RBCs are highly deformable structures that repeatedly pass though narrow capillaries. The adhesion between the membrane bilayer and the cytoskeleton is essential for the mechanical integrity of RBCs, and is provided by linkages between the intracellular domains of membrane proteins and spectrin-based cytoskeletal network. Band 3 and RhAG are important membrane proteins that link to spectrin via ankyrin. Binding of PS to cytoskeletal proteins and the spectrin network also contributes to the mechanical stability and deformability of normal RBCs. Changes in the mechanical integrity and shape of RBCs when converting them into cargo carriers can influence their biodistribution and longevity in circulation. Mechanical characterization of RBC-based constructs is another important area of future research.

Using materials that are already approved for human use by the U.S. Food and Drug administration, such as ICG and MB [132], may help accelerate approval for human studies. Other potential challenges to clinical translation are recently reviewed by Singh et al. and include difficulties with scaling production from a small academic lab to an industrial scale, and thoroughly understanding the potential side effects of a construct, including its cyto-, immuno-, and genotoxicity [133]. In addition, the constructs need to be fabricated in sterile environments using only materials approved for human use (i.e., materials that are not labeled “For Research Use Only”).

Many of the light-based RBC-derived constructs highlighted here have frequently only been used in small-animal models that demonstrate the effectiveness of the constructs for light-based applications. Therefore, there is a large need for more research into the biological interactions of the constructs involving larger animals to not only demonstrate efficacy, but also evaluate biodistribution, immunogenicity, and biocompatibility. In addition, more effort needs to be given to standardizing the methods for fabricating these constructs. Overall, the field of light-based RBC-derived constructs is very promising. New constructs are constantly being developed, increasing the potential for new therapeutics for ultimate clinical translation in a safe and reproducible manner.

## Figures and Tables

**Table 1:** List of acronyms.

Acronym	Definition
AFM	Atomic force microscopy
ALP	Alkaline phosphatase
ALT	Alanine aminotransferase
BSA	Bovine serum albumin
CD	Cluster of differentation
Ce6	Chlorin e6
Cy5	Cyanine 5
DACHPt	1,2-diaminocyclohexane-platinum (II)
DAF	Decay-accelerating factor
DAPI	4′,6-diamidino-2-phenylindole
DiR	1, 1-dioctadecyl-3, 3, 3, 3-tetramethylindotricarbocyanine iodide
DOX	Doxorubicin
DSPE	1,2-distearoly-sn-glycero-3-phosphoethanolamine
EDTA	Ethylenediamine tetraacetic acid
EGs	Erythrocyte ghosts
EPR	Enhanced permeability and retention
FA	Folic acid
Fe_3_O_4_	Iron oxide
FITC	Fluorescein isothiocyanate
HCPT	10-hydroxycamptothecin
H_2_O_2_	Hydrogen peroxide
ICG	Indocyanine green
ICG-EGs	Erythrocyte ghosts loaded with ICG
ICG-nEGs	Nano-sized erythrocyte ghosts loaded with ICG
IG	Immunoglobulin
IL	Interleukin
IR	Infrared
MB	Methylene Blue
MgCl_2_	Magnesium choloride
MOF	Metal–organic framework
MoSe_2_	Molybdenum diselenide
MRI	Magnetic resonance imaging
NaGdF_4_:Yb, Tm	Ytterbium and thulium doped sodium gadolinium fluoride
NaYF_4_:Yb/Er	Ytterbium and erbium doped sodium yttrium fluoride
NHS	N-hydroxysuccinimide
nEGs	Nano-sized erythrocyte ghosts
NIR	Near infrared
NPs	Nanoparticles
PBS	Phosphate buffer saline
PDT	Photodynamic therapy
PEG	Polyethylene glycol
PFC	Perfluorocarbon
PS	Phosphatidylserine
PTT	Photothermal therapy
PWS	Port wine stain
QPs	Quantum dots
RB	Rose bengal
RBCs	Red blood cells
RGD	Arginylglycylasparatic acid
ROS	Reactive oxygen species
SDS-PAGE	Sodium dodecyl sulphate–polyacrylamide gel electrophoresis
TEM	Transmission electron microscope
TiO_2_	Titanium dioxide
TNF	Tumor necrosis factor-alpha
tPA	Tissue plasminogen activator
TPC	5,10,15,20-tetraphenylchlorin
UCST	Upper critical solution temperature
UV	Ultraviolet
UCNPs	Upconversion nanoparticles
ZnF_16_Pc	Zinc hexadecafluorophthalocyanine

**Table 2:** Examples of optical RBC-derived constructs functionalized using DPSE lipid insertion.

Optical Cargo	Targeting Moiety	Target	Reference
Upconversion Nanoparticles (UCNPs)	Folate	Folate receptors	[30]
UCNPs	Triphenylphosphonium cation	Mitochondria	[30]
Gold Nanocages	Anti-epithelial cell adhesion molecule (EpCam) antibodies	EpCam	[38]
UCNPs	Folate	Folate receptors	[42]
Indocyanine Green (ICG)	Anti-human epidermal growth receptor (HER2) antibodies	HER2	[45]
ICG	cRGD pentapeptide	α_v_β_3_ integrin	[48]
ICG-Bovine Serum Albumin (BSA)	RGD peptide	α_v_β_3_ integrin	[62]
ICG-BSA, UCNPs, Rose Bengal (RB)	RGD peptide functionalized UCNPs	α_v_β_3_ integrin	[72]
ICG	Folic acid	Folate receptors	[107]
Prussian Blue (PB) Nanoparticles (NPs)	Hyaluronic acid	CD44 receptors	[110]
Black Phosphorus NPs	YSA peptide	Ephrin-A2 receptors	[121]
ICG	Angiopep-2 peptide	Lipoprotein receptor-related proteins receptors	[122]
Spheric and Cubic Hollow Mesopores PB NPs	Folic acid	Folate receptors	[123]
ICG	Erythropoietin-production human hepatocellular (Eph) B1 receptor ligand binding domain	ephrin-B2 ligands	[124]

## Data Availability

Not applicable.
